# Extracellular DNA metabolism in *Haloferax volcanii*

**DOI:** 10.3389/fmicb.2014.00057

**Published:** 2014-02-20

**Authors:** Scott Chimileski, Kunal Dolas, Adit Naor, Uri Gophna, R. Thane Papke

**Affiliations:** ^1^Department of Molecular and Cell Biology, University of ConnecticutStorrs, CT, USA; ^2^Department of Molecular Microbiology and Biotechnology, George S. Wise Faculty of Life Sciences, Tel Aviv UniversityTel Aviv, Israel

**Keywords:** extracellular DNA, *Haloferax volcanii*, DNA metabolism, Halobacteria, halophiles, archaea, natural competence, archaeal genetics

## Abstract

Extracellular DNA is found in all environments and is a dynamic component of the microbial ecosystem. Microbial cells produce and interact with extracellular DNA through many endogenous mechanisms. Extracellular DNA is processed and internalized for use as genetic information and as a major source of macronutrients, and plays several key roles within prokaryotic biofilms. Hypersaline sites contain some of the highest extracellular DNA concentrations measured in nature–a potential rich source of carbon, nitrogen, and phosphorus for halophilic microorganisms. We conducted DNA growth studies for the halophilic archaeon *Haloferax volcanii* DS2 and show that this model Halobacteriales strain is capable of using exogenous double-stranded DNA as a nutrient. Further experiments with varying medium composition, DNA concentration, and DNA types revealed that DNA is utilized primarily as a phosphorus source, that growth on DNA is concentration-dependent, and that DNA isolated from different sources is metabolized selectively, with a bias against highly divergent methylated DNA. Additionally, fluorescence microscopy showed that labeled DNA co-localized with *H. volcanii* cells. The gene *Hvo_1477* was also identified using a comparative genomic approach as a factor likely to be involved in DNA processing at the cell surface, and deletion of *Hvo_1477* created a strain deficient in the ability to grow on extracellular DNA. Widespread distribution of *Hvo_1477* homologs in archaea suggests metabolism of extracellular DNA may be of broad ecological and physiological relevance in this domain of life.

## Introduction

Extracellular DNA (eDNA) is present in every natural environment, amounting to a global molecular pool measured in gigatons (Dell'Anno and Danovaro, 2005). Beyond its sheer abundance, eDNA is a major component of the microbial ecosystem as a dynamic reservoir of carbon (C), nitrogen (N), phosphorus (P), nucleotides, and genetic information (Dell'Anno and Danovaro, 2005; Corinaldesi et al., 2007, 2008). eDNA is engaged by prokaryotes through often complex endogenous mechanisms, including degradation by unbound and cell-surface-bound secreted nucleases (Heins et al., 1967; Provvedi et al., 2001; Sakamoto et al., 2001; Schmidt et al., 2007; Godeke et al., 2011a), import and export systems mediating natural DNA uptake (Chen and Dubnau, 2004; Maier et al., 2004; Chen et al., 2005; Averhoff, 2009), and as a major component of biofilms (Steinberger and Holden, 2005; Godeke et al., 2011b; Kiedrowski et al., 2011; Gloag et al., 2013).

Hypersaline environments contain some of the highest measured levels of eDNA (Dell'Anno and Corinaldesi, 2004), possibly due to the preservative effect salt has on nucleic acids and other macromolecules (Tehei et al., 2002). Over three hundred micrograms of eDNA per gram of sediment was measured within the first centimeter beneath the water column of a hypersaline lake (Danovaro et al., 2005). However, the mechanisms through which the halophilic microorganisms living in this environment interact, process, and exploit this potential cellular resource are unknown.

Brines above 15% salinity and ranging up to saturation are rich in microbial life and are predominated by euryarchaeal species of the order Halobacteriales, a group commonly known as haloarchaea (Benlloch et al., 2001; Papke et al., 2003, 2004; Pasić et al., 2007; Dassarma and Dassarma, 2008; Andrei et al., 2012). A representative species *Haloferax volcanii* first isolated from Dead Sea sediments in 1975 (Mullakhanbhai and Larsen, 1975) was chosen for this study because it is genetically modifiable, and is a predominant model halobacterial species, and a top archaeal model in general (Bitan-Banin et al., 2003; Allers and Mevarech, 2005; Soppa, 2006; Allers et al., 2010; Blaby et al., 2010; Hartman et al., 2010; Leigh et al., 2011; Atomi et al., 2012). Here we describe an improved understanding of eDNA metabolism in hypersaline environments and the Halobacteria.

## Materials and methods

### Strains and culture media

*Haloferax volcanii* strains (Table 1) were provided by Thorsten Allers of the University of Nottingham, UK and grown in media as previously described (Allers et al., 2004). Hv-YPC contained 144 g NaCl, 21 g MgSO_4_ × 7H_2_O, 18 g MgCl_2_ × 6H_2_O, 4.2 g KCl, 12 mM Tris-HCl pH 7.5, 3.125 ml 1 M CaCl_2_ solution, 1.0 ml trace element solution, 5.0 g yeast extract (Fisher, BP1422), 1.0 g casamino acids (Fisher, BP1424), and 1.0 g peptone per liter (Fisher, BP1420). Casamino acids medium (Hv-Ca) contained all of the above components except for yeast extract and peptone. Starvation medium (used for starve conditions) contained all components in Hv-YPC other than yeast extract, casamino acids, and peptone. Minimal medium (Hv-min) also shared the same concentration of salts and basic constituents, with the addition of 4.25 ml 60% sodium lactate (NaC_3_H_5_O_3_), 5.0 ml 1 M ammonium chloride (NH_4_Cl) solution, and 2.0 ml 0.5 M monopotassium phosphate (KH_2_PO_4_) buffer (pH 7.5). *Escherichia coli* cloning strains and additional bacterial strains used as DNA source and as extracellular nuclease positive control are listed (Table 1) and were grown using standard growth media and conditions.

**Table 1:** **Strains and plasmids**.

**Strain or plasmid**	**Relevant properties**	**References or sources**
**PLASMIDS**		
pTA131	Integrative vector based on pBluescript II; AmpR with BamHI-XbaI fragment from pGB70 containing *pfdx-pyrE2*	Allers et al., 2004
pTA409	Shuttle vector based on pBluescript II, with *pyrE2* and *hdrB* markers and *ori-pHV1/4* replication origin	Hölzle et al., 2008
pKD131_Δ1477	pTA131 with HindIII-NotI fragment containing *Hvo_1477* flanking regions for pop-in pop-out gene deletion	This study
pKD409_1477c	pTA409 with BamHI-EcoRI fragment containing *Hvo_1477* and native upstream promoter	This study
**STRAINS**		
*E. coli*		
DH5α	Used for subcloning and as source of methylated *E. coli* DNA	Invitrogen, 18263012
K12	dam^−^/dcm^−^ strain; used for source of unmethylated DNA	New England Biolabs, C2925I
*H. volcanii*		
DS2	Wild-type	Mullakhanbhai and Larsen, 1975
H26	Δ *pyrE2*	Allers et al., 2004
Δ *Hvo_1477*	*Hvo_1477* deletion in H26 background	This study
Δ *Hvo_1477*c	Δ *Hvo_1477* with pKD409_1477c	This study

All media used in growth experiments with variable C, N and P availability were derivates of Hv-min. Conditions denoted as CNP contained C, N and P sources (NaC_3_H_5_O_3_, NH_4_Cl, and KH_2_PO_4_, respectively) at the same final concentration as Hv-min medium above. Additional conditions/media derivatives lacked either sodium lactate, ammonium chloride, and/or potassium phosphate (e.g., NP medium contains NH_4_Cl, and KH_2_PO_4_, but no carbon source).

### DNA extraction and purification

The purity and integrity of supplemented high molecular weight (HMW) DNA was a primary consideration. Chromosomal DNA for supplementation was isolated from source species through standard lysis methods, followed by proteinase K digestion and ethanol precipitation. DNA was further purified through multiple phenol/chloroform/isoamyl alcohol (pH 8.0) extractions until no protein-rich interphase was present, followed by three chloroform/isoamyl alcohol extractions to remove trace phenol and an additional ethanol precipitation. DNA was then dissolved in 10 mM Tris-Cl solution (pH 8, in DNA-grade water) and passed through a mini polyacrylamide gel filtration spin column according to the manufacturer protocol (Bio-Rad Bio-Spin P-30, in Tris buffer, 732–6231) to remove small molecules including free nucleotides and oligonucleotides <20 bp in length. Purified DNA was used fresh for growth experiments to reduce subsequent hydrolysis, and was sterilized with a 0.22 μm filter prior to supplementation. RNA was degraded within DNA samples using RNase I (Thermo, FEREN0601) according to manufacturer's protocol, followed by heat inactivation, ethanol precipitation, and resuspension. DNase digested DNA used in growth experiments was digested with DNaseI (Invitrogen, 18068–015) for 12 h at 37°C. Herring sperm DNA was from Promega (D1811).

DNA concentration and purity was determined using a Nanodrop ND-1000 (OD _260 nm_/OD _280 nm_ = 1.8) or a Qubit 2.0 fluorometer (Q32866) with dsDNA High Sensitivity kit (Invitrogen, Q32854). HMW DNA was visualized on agarose gels prior to supplementation.

### eDNA metabolism studies

**Figure 1:**
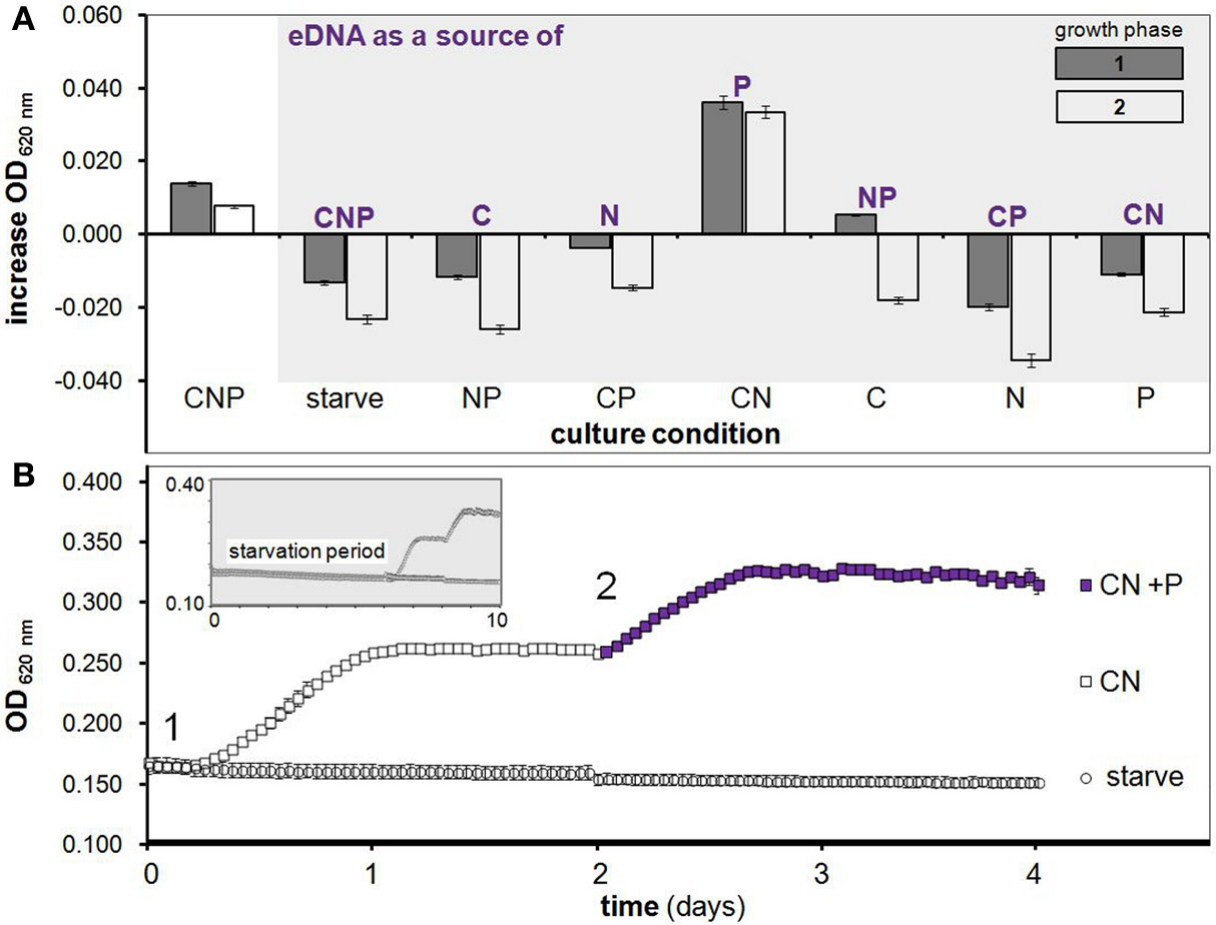
***H. volcanii* growth on eDNA as a C, N and/or P source**. **(A)** Increase in OD during two growth phases after starvation (as in growth curve below) with eDNA supplementation in Hv-min medium containing all possible combinations of C, N and P. **(B)** After a 6 day starvation period (shown in smaller gray box) cultures were supplemented with C and N (squares) or a negative control starvation solution (circles) at *T* = 0 days (point 1) with OD measurements taken every hour. After one round of growth to stationary phase, KH_2_PO_4_ (purple filled squares) was added to CN cultures as a P source at point 2 and induced a second growth phase. Error bars are SD of replicate cultures.

For all growth experiments a minimum of three replicate cultures per condition began with an individual *H. volcanii* DS2 colony grown to mid-exponential phase (OD _600 nm_ ~0.4) at 42°C in liquid Hv-YPC, washed three times with starvation medium, and diluted in medium specific to the experiment (e.g., starvation medium or Hv-min derivative). When cultures were starved, starvation occurred at 42°C for a ≥5 day period prior to supplementation to allow for depletion of internal nutrient stores (particularly phosphorus, see Figure 1B).

Growth on eDNA was tested in several ways: 200 μl of culture within 96-well plates (sealed with transparent plastic film to avoid evaporation and salt precipitation), 5 ml of culture within 50 ml plastic culture tubes, 10 ml of culture in glass anaerobic culture tubes (with rubber stoppers and aluminum seals, head-space displaced with N_2_ and supplemented with 50 mM sodium nitrate), and 20 ml of culture in 125 ml cell culture flasks (baffled and unbaffled). All experiments were conducted at 42°C. Cultures were shaken at 180 rpm, other than anaerobic tubes (not shaken).

For experiments with either eDNA and/or C, N, and P sources added after a starvation period (no C, N or P), supplementation occurred through a 10% v/v addition of purified DNA solution, and/or 10× sodium lactate, ammonium chloride or monopotassium phosphate solution (in DNA solvent, 10 mM Tris-Cl, pH 8.0), or negative control solution (10 mM Tris-Cl, pH 8.0). Optical density measurements of replicate cultures were taken on a shaking and incubating microplate reader (ThermoFisher Multiskan FC, measurement filter 620 nm) or a Biorad SmartSpec Plus (600 nm) over a 5–10 day period (depending on length of starvation, or induction of several growth phases as in Figure 1). All OD values from culture tube or flask experiments were measured using the Biorad SmartSpec Plus and are greater than identical samples read in 96-well microplates on Multiskan FC; this is caused by the difference in measured wavelength (600 and 620 nm) and a volume added to each microplate well (200 μl) less than the full path length (1 cm). Therefore, there are non-random differences in OD values between experiments measured by OD at 600 and 620 nm.

Viable cell count experiments were conducted within 96-well microplates. Triplicate *H. volcanii* DS2 cultures were starved of KH_2_PO_4_ for 5 days in Hv-min CN medium after which samples were removed at indicated time points. The *T* = 0 sample was taken after the addition of DNA solvent or unmethylated *E. coli* DNA to a final concentration of 500 μg/ml, and optical density was monitored simultaneously. After 4 days the stationary phase sample was taken and cell titers were quantified through a serial dilution of each culture and plating for colony forming units (CFUs) on Hv-YPC medium.

Where significant difference is noted between conditions for growth experiment results, significance of difference between data series was determined by One-Way ANOVA test of the mean for each replicate set (*n* ≥ 3 biological replicates). A significant difference between means was defined as a *P* ≤ 0.05, while no significant difference between two means was defined as a *P* ≥ 0.05. Where increase in OD is shown, average increase in OD was calculated by subtracting the initial OD value at time zero, or OD of control culture (e.g., value for CN medium alone from value for CN +eDNA), from the final value achieved after incubation for each replicate.

### Assessment of extracellular DNase activity

Conditioned media (CM) was harvested from *H. volcanii* DS2 and *Staphylococcus aureus* (as a positive control) by centrifuging exponential phase (OD _600 nm_ ~0.6) cells grown in rich medium and passing the supernatant through a 0.22 μm filter. Medium had been previously inoculated with a single colony. CM harvested from the two species was supplemented with 30 μg of unmethylated pTA131 plasmid DNA (final concentration 150 ng/μl), and incubated for 12 h at 37°C. During this time, secreted nuclease in the CM is exposed to and may degrade high molecular weight DNA fragments, resulting in smearing of lower molecular weight DNA detectable on an agarose gel stained with ethidium bromide. The DNase I positive control digestion was performed according to manufacturer protocol (Invitrogen, 18068–015).

### Fluorescence microscopy

One microgram of unmethylated *E. coli* DNA as prepared for growth studies was first digested for 10 min with DNase I (Invitrogen, 18068–015) to increase reaction efficiency and probe fragmentation and labeled with the Ulysis Alexa Fluor® 488 Nucleic Acids Labeling Kit (Molecular Probes, U-21650). The labeling reaction occurs during an 80°C incubation step, creating an irreversible complex between the Alexa Fluor® fluorophore and guanine and adenine bases. Labeled DNA was purified from un-reacted probe using mini-gel filtration spin columns (Bio-Rad Bio-Spin P-30, in Tris buffer, 732–6231) as recommended.

*H. volcanii* DS2 cells were grown to mid-log phase (OD 0.4) in Hv-YPC medium, pelleted, washed three-times and resuspended in a basal salts medium (Hv-starve). Cells were incubated with freshly labeled and purified DNA at a final concentration of 10 ng/μl for 1 h at 42°C, after which they were pelleted once again and suspended in Hv-starve medium to remove excess probe. Preparations of live cells were visualized immediately using a Nikon ECLIPSE TE-300 inverted fluorescence microscope. Photographs of labeled cells viewed at 600× total magnification were collected under white light and with excitation at 488 nm (pseudocolored green). Cells did not autofluoresce at the tested excitation wavelength as verified by no detectable signal in identically prepared unlabeled cells.

### Identification of putative DNA metabolism gene Hvo_1477 and related genes

The Hvo_1477 protein (YP_003535526) was identified as a putative membrane-bound nuclease involved in DNA metabolism through a BLASTP search (Altschul et al., 1990) of the *H. volcanii* genome (Hartman et al., 2010) using known bacterial proteins (Table 2) as queries. Hvo_1477 was targeted as a homolog of bacterial nuclease YokF (YP_007534137). Clusters of Orthologous Groups or COGs (Tatusov, 1997), protein domains, and protein superfamilies (Gough and Chothia, 2002; Sigrist et al., 2013) were identified using the MicrobesOnLine portal (Dehal et al., 2010).

**Table 2:** **Hvo_1477 protein homologs with known functions**.

**Species**	**Name**	**Accession numbers**	**Functions and features**	**References**	**BLAST *e*-value**
*Bacillus subtilis*	YokF	YP_007534137	Surface-bound nuclease, lipoprotein/involved in eDNA metabolism	Sakamoto et al., 2001	2e-22
	YhcR	EME06910	Sugar-non-specific surface-bound endonuclease, sortase substrate	Oussenko et al., 2004	4e-12
	YncB	YP_007533717	Nuclease, YokF paralog	Sakamoto et al., 2001	1e-27
*Staphylococcus* spp.	Nuc	BAH56528	Secreted nuclease, conditionally surface-bound/ modulator of biofilm formation	Chesneau and El Solh, 1994; Kiedrowski et al., 2011	5e-25
					

The phylogenetic tree of archaeal YokF/Hvo_1477 homologs was created using Seaview (Gouy et al., 2010). All homologs shown have an *E*-value of 1e-10 or lower in a pairwise BLASTP with YokF and were aligned with Clustal Omega (Sievers et al., 2011). The tree was constructed using PhyML (Guindon and Gascuel, 2003).

### Gene deletion and complementation

Deletion of *Hvo_1477* was carried out using the pop-in pop-out method as previously described (Bitan-Banin et al., 2003; Allers et al., 2004). Briefly, the upstream flanking region of *Hvo_1477* was amplified and restriction sites were incorporated into PCR products within primers (Table 3). The PCR products were purified using a Qiagen PCR purification kit (Qiagen, 28106) and digested with EcoRI (enzymes were purchased from New England Biolabs). The flanking region products were then purified using a Qiagen gel purification kit (Qiagen, 28706) and ligated with T4 ligase (Promega, M1804). The final product was verified using the forward primer of the upstream flanking regions and the reverse primer of the downstream flanking region (Table 3). Plasmid pTA131 (Table 1) and ligated flanking regions were digested with HindIII and NotI and the digested products were gel purified and ligated. The ligated plasmid (pTA131_ 1477del) was transformed into competent *E. coli* cells from New England Biolabs (Table 1), grown on LB-ampicillin plates and extracted from isolated ampicillin resistant colonies. Competent cells of *H. volcanii* strain H26 were made and transformed with the extracted plasmid via the standard polyethylene glycol method (Charlebois et al., 1987; Dyall-Smith, 2009) and plated on Hv-Ca medium for pop-in. Pop-in colonies were selected through colony PCR and plated on Hv-Ca with 5-FOA (Zymo Research, F9003) to counter-select for pop-outs. Final deletion mutants were identified through a colony PCR screen of 5-FOA resistant colonies.

**Table 3:** **Oligonucleotide primers used**.

**Primer**	**Sequences (5′ −3′)^a^**	**Properties**
Hvo_1477FR1_F	TTTAAGCTTCGTGCGCCCGATTTCCTTCT	*Hvo_1477* deletion, upstream region external, HindIII site
Hvo_1477FR1_R	TTTGAATTCTCGATTCACCGTTAGTCAGGG	*Hvo_1477* deletion, upstream region internal, EcoRI site
Hvo_1477FR2_F	AAAGAATTCCGACGAAGTGCTCGCGTACA	*Hvo_1477* deletion, upstream downstream region internal, EcoRI site
Hvo_1477FR2_R	TTTGCGGCCGCCGAGATGCGCGGCGAGGT	*Hvo_1477* deletion, downstream region external, NotI site
Hvo_1477P_F	AAAGGATCCGTTCCATTAAAAGGTTTCTGGT	Forward complementation, with native promoter, BamHI site
Hvo_1477_R	AAAGAATTCCAGTGTCTCCCCGAACAGCGG	Complementation, reverse, EcoRI site

a Restriction endonuclease sites are underlined.

For complementation plasmid construction, the native promoter for *Hvo_1477* was predicted with the Neural Network Promoter Prediction site (http://www.fruitfly.org/seq_tools/promoter.html) and primers were constructed (Table 3) to include this region (beginning 125 bp from the start codon of *Hvo_1477*). The product containing *Hvo_1477* and promoter was ligated into pTA409 after digestion and gel purification of insert and plasmid with BamHI and EcoRI, creating the plasmid pKD409_1477c (Table 1). The product was transformed into competent *E. coli* cells and colonies were selected and confirmed using PCR. Amplified product was purified, transformed into *H. volcanii* Δ *Hvo_1477* cells (Table 1) and then plated for selection on Hv-Ca plates. Colonies that had regained uracil prototrophy and grew on Hv-Ca were grown in liquid medium and the final complemented strain (Δ*Hvo_1477*c, Table 1) was confirmed using colony PCR.

## Results

### eDNA metabolism and primary role as a phosphorus source

An ability to utilize eDNA to drive metabolic growth in *H. volcanii* was observed using several experimental platforms and methods. We first used 96-well microplates as a routine method for monitoring growth through optical density of replicate cultures, and discovered an increase in OD of *H. volcanii* cultures after supplementation with RNase treated, freshly precipitated and highly purified HMW double-stranded DNA (as in Figure 1). Initial experiments were followed by additional microplate-based studies whereby all possible combinations of typical C, N and P sources were supplemented after a starvation period (aimed at depletion of intracellular stores).

This next phase of starvation experiments lead to the principle findings that (i) *H. volcanii* stores phosphorus intracellularly, and (ii) eDNA is utilized primarily as a source of phosphorus. Internal P storage is demonstrated by the observation that cells starved of C, N and P for 6 days and then supplemented with C and N but not P were able to reproduce through approximately one growth phase (Figure 1B). A second growth phase was then induced in these same replicate cultures by the addition of KH_2_PO_4_ alone, further indicating that cessation of growth was indeed due to P limitation (Figure 1B, purple squares). Likewise, eDNA's role as a P source is demonstrated by the observation that eDNA supplementation in starved cultures led to a significant growth advantage only when cultures were provided with C and N (CN medium, Figure 1A)–further verified with a second addition of eDNA after CN culture cells had reached the stationary phase—which caused a second phase of exponential growth, again only in CN medium (Figure 1A, white bars). DNaseI digested DNA was also tested and led to growth in CN medium equal to that of undigested DNA (data not shown), indicating *H. volcanii* can utilize nucleotides and small olgionucleotide products in addition to HMW DNA. While microplate experiments are useful for high-throughput assays encompassing many conditions, concerns regarding growth limitation due to small culture volume and oxygen availability led us to validate observed trends using several independent culturing methods and conditions.

**Figure 2:**
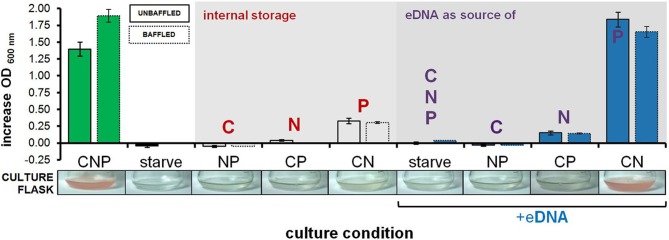
**Growth on eDNA as a C, N and P source in culture flasks**. *H. volcanii* cultures were grown in Hv-min derivatives deficient in C, N, or P (NP, CP, CN, respectively) with and without eDNA supplementation. Bars represent increase in optical density after 60 h of incubation in unbaffled (solid lines) and baffled (dashed lines) flasks. A photograph of a representative replicate culture flask is also shown, with characteristic red color of halobacterial cells in dense cultures in the CNP control and CN + eDNA flasks. Error bars represent standard deviation of replicate cultures.

**Figure 3:**
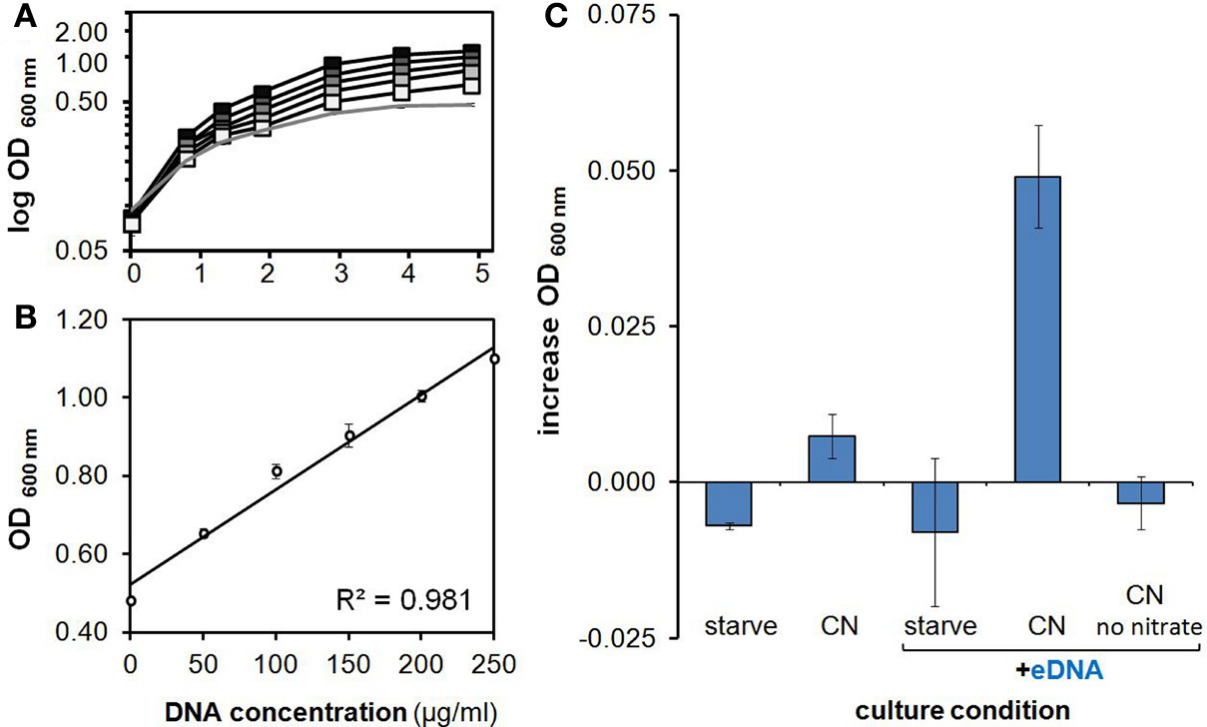
**Characterization of growth on eDNA as a phosphorus source**. **(A)** Growth on eDNA as a P source is concentration dependent. *H. volcanii* DS2 cultures in minimal medium lacking a phosphorus source were provided with unmethylated *E. coli* DNA at final concentrations of 50, 100, 150, 200, and 250 μg/ml (gray squares, increasing darkness). **(B)** Optical density achieved after 5 days of incubation at increasing DNA concentrations. **(C)** Growth on eDNA after 48 h during anaerobic nitrate respiration. *H. volcanii* DS2 cultures in minimal medium lacking a phosphorus source were prepared and grown under anaerobic conditions with unmethylated *E. coli* DNA at a final concentration of 200 μg/ml. All cultures other than the no nitrate eDNA control contained 50 mM sodium nitrate. Errors bars represent standard deviation of replicates.

Further OD-based studies included a dose-dependence experiment, in addition to culturing in larger volumes within baffled and unbaffled flasks (Figure 2), and in culture tubes during both aerobic (Figures 3A,B) and anaerobic conditions (Figure 3C). As expected, a linear relationship between increasing eDNA concentration and OD _600 nm_ was measured during growth in CN medium: with absorbance readings reaching 129% above control values at 250 μg/ml (Figure 3B). A scaled-up experiment with 20 ml of culture grown in culture flasks (100× greater volume than microplate wells) was conducted with OD readings taken after eDNA or DNA solvent was supplemented in NP, CP or CN medium inoculated with starved *H. volcanii* cells. As in microplate-based experiments, growth in each medium type without eDNA is indicative of a capacity for internal storage of the missing macronutrient. Relatively weak but significant growth (*P*-value of 0.002 when compared to starvation cultures) without eDNA was only measured in CN medium, affirming internal P storage, and suggesting insufficient internal C or N stores capable of driving cellular division (Figure 2). Also consistent with microplate experiments, eDNA supplementation led to a large increase in OD only in CN medium, and a small but significant increase in CP medium, confirming the use of eDNA as a P source and suggesting a role as a weak nitrogen source (Figure 2). Cell cultures grown in anaerobic tubes during nitrate respiration were also able to utilize eDNA as a P source (Figure 3C).

Viable cell counts also verified growth on eDNA as a P source. Averaged CFUs at stationary phase for a culture starved of P (through growth in CN media, as in Figure 1B) within a microplate were over seventy times greater with eDNA supplementation as compared to control cultures (DNA solvent alone) (Table 4). The optical density-based growth curve for this culture indicated approximately one doubling during this same period (Table 4), typical of most eDNA supplementation experiments described here. This indicates that while OD measurements are useful for revealing overall trends, viable cell numbers are underestimated, likely due to difference in light scattering properties such as cell shape, size, and intracellular composition.

**Table 4:** **Viable cell count of phosphorous-starved DNA supplemented *H. volcanii* DS2 cultures**.

	***T*** = **0 (lag phase)**		**4 days (stationary phase)**		**Change in average CFU/ml**
	**CFU/ml**	**OD _620_ nm^a^**	**CFU/ml**	**OD_620_ nm**	
DNA solvent	4.47 × 10^5^ ± 4.04 × 10^3^	0.1708 ± 2.30 × 10^−3^	9.26 × 10^4^ ± 1.33 × 10^3^	0.1648 ± 1.10 × 10^−3^	2.07-fold decrease
500 μg/ml DNA	3.93 × 10^5^ ± 2.73 × 10^3^	0.1657 ± 2.22 × 10^−3^	2.82 × 10^7^ ± 1.20 × 10^5^	0.3015 ± 4.45 × 10^−3^	71.8-fold increase

a Corresponding optical density at time of sampling. Error shown is SD of counts from triplicate cutures.

### Selective metabolism of available eDNAs

**Figure 4:**
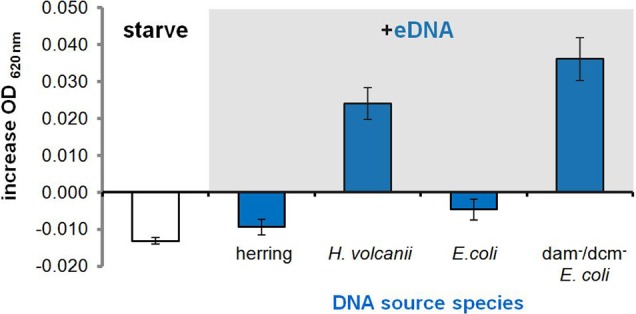
**Selective metabolism of available eDNAs**. Average increase in OD of P-starved *H. volcanii* cultures in CN medium 16 hours after supplementation with 250 μg/ml eDNA isolated from different source species. Error bars are standard deviation of replicates.

Our first observation of growth on eDNA occurred when supplementing *H. volcanii* with its own genomic DNA (i.e., conspecific DNA). However, we soon noticed an inability to metabolize certain DNA types when we attempted to grow *H. volcanii* on eDNA extracted from other DNA sources. This began with an inability to utilize herring sperm DNA and *E. coli* DNA (no growth advantage in CN media, Figure 4).

We then tested additional DNA types in order to identify any features or properties of available eDNA that could be discriminated by *H. volcanii* cells and any associated molecular components involved in DNA metabolism. After a bioinformatic search for DNA uptake signals like those found throughout the genomes of many competent Gram negative bacteria (Redfield et al., 2006) produced no putative short hyper-represented motifs, we supplemented *H. volcanii* cultures with eDNA extracted from *Micrococcus luteus*, a divergent bacterial species with a high G-C content similar to that of the *Haloferax volcanii* genome (Hartman et al., 2010), and again observed no growth (data not shown). This decreased the likelihood that selectivity was primarily due to an uptake signal sequence feature or G-C content and we moved on to chemical modification of eDNA by methylation.

**Figure 5:**
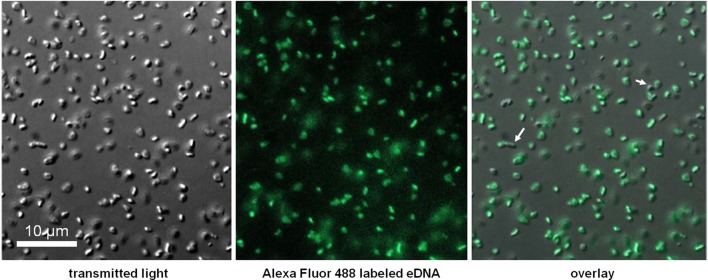
**Co-localization of labeled eDNA and *Haloferax volcanii* cells**. Unmethylated *E. coli* DNA was labeled with Alexa Fluor 488, incubated with starved cells, and visualized at 600× using an epifluorescence microscope. Auto-fluorescence was not detected at excitation wavelength.

DNA extracted from an *E. coli* K12 strain with *dam* and *dcm* DNA methyltransferases deleted (Table 1) was tested, and we observed that along with conspecific DNA, unmethylated *E. coli* DNA led to a significant increase in OD at stationary phase (Figure 4). An *E. coli* strain with a single DNA methylation gene deleted (*dam*) was also tested and showed significant growth between that of fully methylated (DH5α, no growth) and unmethylated DNA (data not shown). Unmethylated *E. coli* DNA fragments were also labeled with a fluorescent probe and incubated with *H. volcanii* in liquid culture to test for association of cells with eDNA. A majority of cells as visualized under white light (first panel) co-localized with strong signal from labeled eDNA (Figure 5, third panel), while some visible cells in the focal plane appear not to co-localize with eDNA (Figure 5, third panel, white arrows).

### Screens for hydrolase activity within conditioned medium indicate absence of unbound secreted nuclease

**Figure 6:**
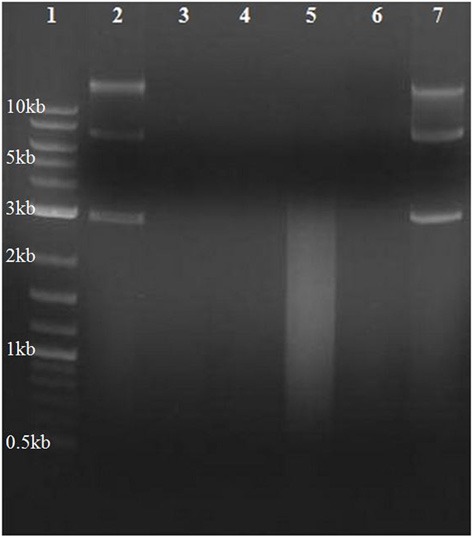
**Assay for secreted unbound nuclease activity in *H. volcanii***. Conditioned media was harvested from *H. volcanii* and *S. aureus* cultures, supplemented with plasmid DNA, and incubated at 37°C. Gel electrophoresis of DNA samples recovered after 12 h of incubation with CM is shown. Lane numbers are (1) 2-log DNA ladder (NEB N3200L), (2) DNA incubated with unconditioned LB medium, (3) DNA incubated with DNase I, (4) *S. aureus* CM, (5) *S. aureus* CM incubated with DNA, (6) *H. volcanii* CM, and (7) *H. volcanii* CM incubated with DNA.

DNase activity from CM was assayed to evaluate the presence of hydrolytic enzyme secreted into the environment during growth. CM from the nuclease secreting bacterial species *S. aureus* (Figure 6, lane 5) produced the expected smear of DNA fragments ranging from ~2000 to 200 bp, with none of the original bands remaining. DNA within *H. volcanii* CM remained intact (Figure 6, lane 7), as in lane 2 in which DNA was added to non-conditioned medium, indicating an absence of evidence for eDNA degradation in *H. volcanii* CM. Previous studies have also reported a lack of secreted nucleases in haloarchaeal species (Ventosa et al., 2005).

### Hvo_1477 is important for DNA metabolism in *H. volcanii*

**Figure 7:**
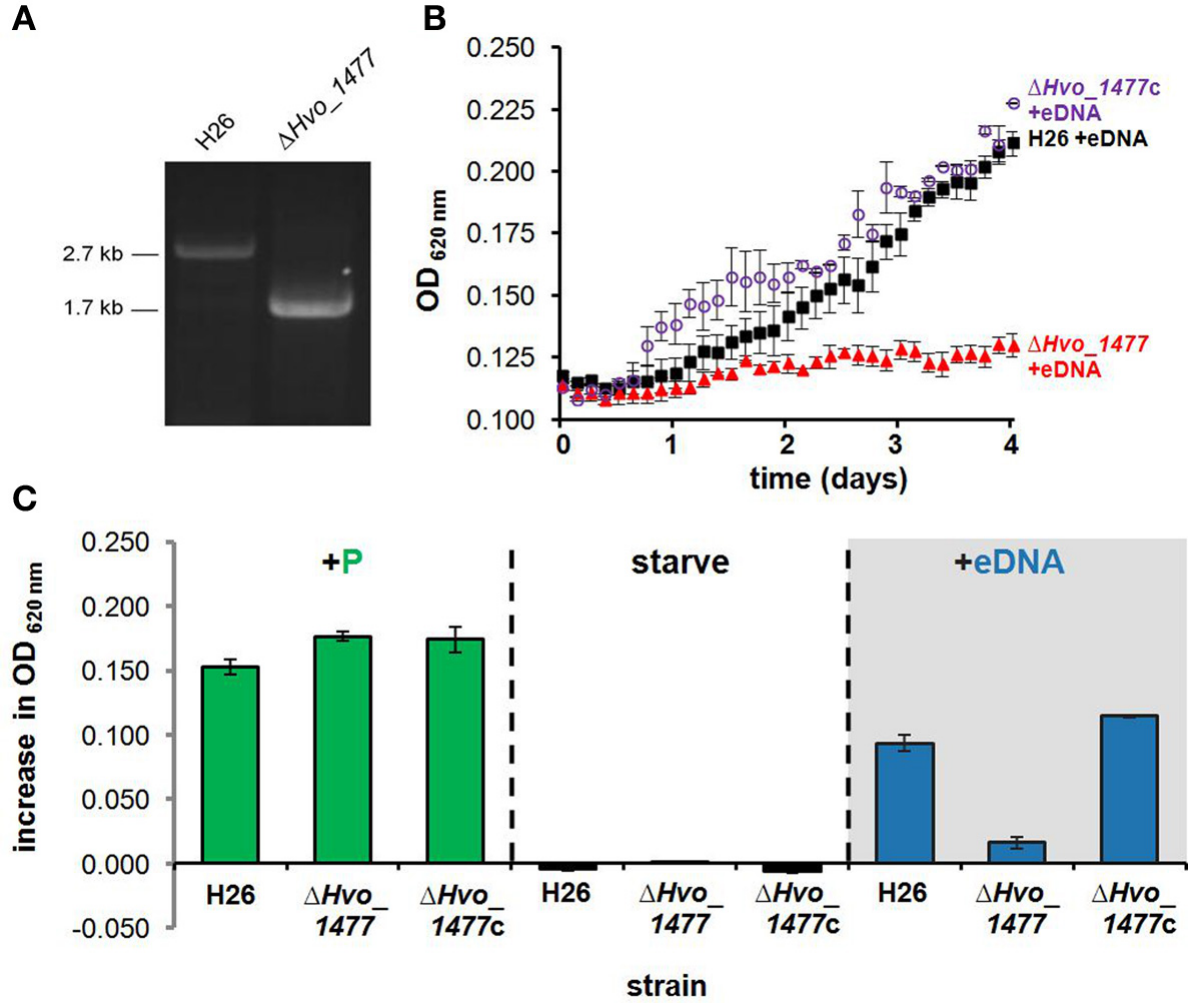
***Hvo_1477* is required for growth on DNA**. **(A)** Deletion of chromosomal gene *Hvo_1477* in *H. volcanii* strain H26. PCR amplification of H26 and Δ *Hvo_1477* template DNA using the forward primer for the upstream *Hvo_1477* flanking region, and the reverse primer of the downstream flanking region (Hvo_1477FR1_F and Hvo_1477FR2_R, Table 3). **(B)** Growth with eDNA in CN media for H26 (parental strain, black filled squares), Δ *Hvo_1477* (red filled triangles) and Δ *Hvo_1477* with pKD409_1477c complementation plasmid (Δ *Hvo_1477*c, purple circles). OD was measured every 3 h within a shaking and incubated 96-well plate reader. **(C)** Increase in optical density 96 h after supplementation with KH_2_PO_4_, DNA solvent (continued starvation), or eDNA. Error bars represent standard deviation of triplicate cultures.

Deletion of *Hvo_1477* diminished growth on DNA (Figure 7). This phenotype was confirmed by complementation with plasmid pKD409_1477 (Table 1) containing *Hvo_1477* and its native promoter, and resulted in the restoration of growth on DNA to levels slightly greater (*P* = 0.049) than that of the parental strain (H26, Figure 7), possibly due to multiple copies of the plasmid. No additional phenotype for the Δ *Hvo_1477* strain has been observed at the time of publication; growth rates in minimal medium (Hv-min) with sodium lactate as a carbon source are equal to that of H26 (Figure 7, +P).

**Figure 8:**
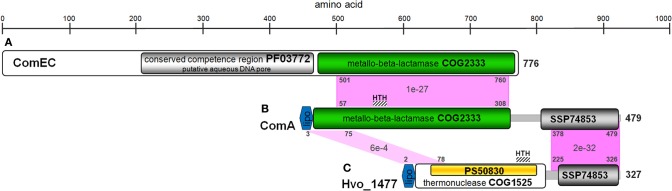
**Protein sequence and domain structure similarity of *B. subtilis* ComEC, *Halobacterium* sp. NRC-1 ComA, and *H. volcanii* Hvo_1477**. Homologous regions between proteins are shown in pink with representative positions and *E*-values. Domains and features listed (Table 5) are also shown. **(A)** NP_390435, **(B)** NP_395842, **(C)** YP_003535526.

**Figure 9:**
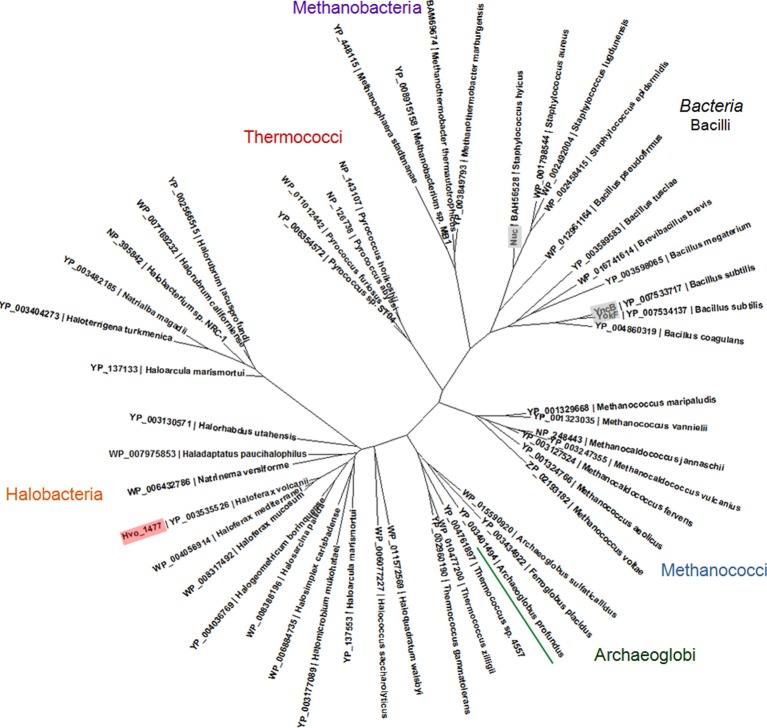
**Phylogenetic tree of archaeal YokF/Nuc protein homologs**. A protein alignment was created with Clustal Omega (Sievers et al., 2011) and used to create a maximum likelihood tree using PhyML (Guindon and Gascuel, 2003). Proteins included in alignment were identified in a BLASTP search of the non-redundant protein database and have an *E*-value of 1e-10 or lower in a pairwise BLASTP with YokF. Accession numbers and species names are shown; bacterial proteins with known functions (see also Table 2) are labeled gray.

Hvo_1477 is a 327 aa protein that has a predicted size of 34.1 kDa, several annotated sequence features (Table 5), and is homologous to known bacterial extracellular nuclease proteins YokF, YhcR, and YncB of *Bacillus subtilis*, and Nuc of staphylococcal species (Chesneau and El Solh, 1994) (Table 2). While the *Hvo_1477* gene and associated haloarchaeal homologs are annotated as competence-like protein-encoding genes or *comA*, there is no homology between the Hvo_1477 protein sequence and bacterial competence protein orthologs ComA/ComEC, which contain multiple membrane-spanning regions and are known to form an aqueous DNA pore during natural DNA uptake (Facius and Meyer, 1993; Draskovic and Dubnau, 2005) (Figure 8). The basis for this annotation may be that some halobacterial homologs of Hvo_1477 (including *Halobacterium* sp. NRC-1, as shown in Figure 8) are larger proteins containing an additional metallo-beta-lactamase domain (COG 2333) that does share a region of similarity with bacterial ComA/ComEC. These larger haloarchaeal bacterial nuclease homologs clustered together in the phylogenetic tree of Hvo_1477 homologs (Figure 9, group 2), and some species such as *Haloarcula marismortuti* have both the smaller thermonuclease containing (see Figure 9, group 1), and larger metallo-beta-lactamase containing version. However, homology of group 2 proteins (Figure 9) with ComEC/ComA appears to be based on only a single shared domain (COG 2333): all haloarchaeal homologs are missing the important putative DNA pore domain or “conserved competence region” (Figure 8).

**Table 5:** **Annotated features/domains of Hvo_1477**.

**ID**	Database^*^/domain ID	**Start/end**	**Description**
PROKAR_LIPOPROTEIN	PS51257	1/20	Lipobox motif
TNASE_3	PS50830	67/92	Thermostable nuclease domain
Micrococcal nuclease	COG1525	23/215	Micrococcal nuclease domain
Staphylococal nuclease	SSF50199	55/203	Staphylococcal nuclease superfamily
Helix-turn-helix	HTH	171/192	DNA binding motif
Thermonuclease active site	PS01123	67/92	Active site of thermonuclease
Lamin A/C globular tail	SSF74853	236/326	No putative function

* Database abbreviations: PS, PROSITE Database (Sigrist et al., 2013); COG, Cluster of Orthologous Groups (Tatusov, 1997); SS, SCOP Superfamily Database (Gough and Chothia, 2002).

## Discussion

The discovery of an ability to metabolize HMW eDNA in the Halobacteria is a central finding of this work. While bacterial species are known to use eDNA as a nutrient source (Finkel and Kolter, 2001; Sakamoto et al., 2001; Palchevskiy and Finkel, 2006; Lennon, 2007; Pinchuk et al., 2008; Mulcahy et al., 2010), here we report this capacity in an archaeon. Most bacterial species known to use DNA as a nutrient metabolize DNA as a source of C, N and/or P; here we show that *H. volcanii* uses eDNA almost exclusively as a source of phosphorus (Figures 1, 2).

Our experimental demonstration of DNA metabolism as a P source in *H. volcanii* adds to previous reports showing that (i) eDNA concentrations are exceptionally high in hypersaline sample sites and (ii) organisms living in hypersaline environments are often limited by phosphorus (Oren and Shilo, 1982; Oren, 1983; Ludwig et al., 2006). We therefore propose that nutritional DNA uptake may be a primary mechanism through which haloarchaeal species obtain phosphorus and that DNA is likely a major currency of P exchange and storage in hypersaline environments. DNA is indeed a P-rich molecule (10% by weight), and has been shown to account for over 40% of P cycling in some environments (Dell'Anno and Danovaro, 2005; Corinaldesi et al., 2007, 2008). Interestingly, because *H. volcanii* is highly polyploid, intracellular DNA stores may also be important for biogeochemical systems in the environment (Soppa, 2013). Furthermore, the distribution of *Hvo_1477* homologs throughout the Euryarchaeota (Figure 9) suggests DNA metabolism could be an important physiological ability relevant in many species and ecosystems.

We also discovered a bias in metabolism toward conspecific and unmethylated eDNAs, whereby highly divergent eDNA is only utilized when unmethylated (Figure 4). Because *H. volcanii* methylates its own DNA (Hartman et al., 2010), we suggest available eDNAs are processed through recognition of methylation patterns. This is the first report demonstrating the importance of methylation for eDNA discrimination and the extent of this characteristic among other prokaryotes is unknown. However, the presence of such a system for discrimination of eDNA offers a possible explanation to the finding that eDNA accumulates and remains preserved in environments despite high overall levels of DNase activity (Corinaldesi et al., 2008). High concentrations of eDNA found in a particular environment may reflect the inability of all organisms living there to utilize the available DNA because they cannot process and/or import it. For instance, there are many bacterial and eukaryal cells that live in hypersaline environments, and their DNA would not be methylated in a manner that *H. volcanii* can recognize and utilize.

*Hvo_1477* is the first eDNA processing/uptake related gene identified in an archaeon–a starting point toward understanding an archaeal eDNA degradation mechanism and associated phenotypes. Hvo_1477 is not a ComA/ComEC homolog, but is instead a putative lipoprotein with a thermonuclease domain (Tables 2, 5, Figures 8, 9). Lipoproteins are secreted and attached to the cell surface in both bacterial and archaeal species (Szabo and Pohlschroder, 2012) and surface-bound nucleases in bacteria such as the YokF-related *B. subtilis* Hvo_1477 homologs listed in Table 2 (Sakamoto et al., 2001; Oussenko et al., 2004) and ExeM (not a Hvo_1477 homolog) in *Shewannela* species (Godeke et al., 2011a) are known to be involved in DNA metabolism. Deleting *yokF* and its paralogs (e.g., *yncB*) in *B. subtilis* also greatly reduced but did not abolish growth levels on eDNA (Sakamoto et al., 2001). The use of DNA as a nutrient is considered a form of natural DNA uptake or natural competence (Finkel and Kolter, 2001) (NC); however, in strict terms, NC is defined by internalization of intact DNA fragments and by the presence of a complex molecular machine responsible for DNA binding, processing, and internalization (Chen and Dubnau, 2004). At this point, we have not conclusively demonstrated that HMW eDNA is imported across halobacterial membranes.

It remains unclear in *H. volcanii* whether Hvo_1477 is associated with additional surface or transmembrane proteins and if eDNA is imported as HMW DNA into the cell. Other bacterial surface-bound nucleases, including EndA of *Streptococcus* species and NucA in *Bacillus* species are responsible for processing eDNA prior to internalization by additional proteins (Provvedi et al., 2001; Chen and Dubnau, 2004). It is possible then that Hvo_1477 also acts as part of an unknown archaeal DNA uptake complex. The observation that *H. volcanii* is biased against most sources of DNA it can utilize for metabolism suggests that HMW DNA is indeed moved across the membrane and into the cell; if DNA hydrolysis occurred extracellularly, and only nucleotides were imported for growth, it is difficult to explain why cells would reject most DNA sources and undergo starvation. Fluorescence microscopy experiments revealed that labeled eDNA co-localizes with *H. volcanii* cells (Figure 5), consistent with the assumption that HMW DNA associates with the cell during a multi-part process of DNA processing and metabolism. Because not all cells co-localized with DNA, it is possible that a fraction of cells within a given population do not express DNA binding factors (i.e., regulated expression) and are unable to associate with eDNA. The identification of protein-protein interactions, regulation, and dynamics of eDNA processing at the cell surface (including cellular binding assays with multiple DNA types), and further biochemical characterization of Hvo_1477 are necessary for further insight.

Drawing from studies of surface-associated nucleases in bacterial species, it seems likely that *Hvo_1477* has additional important phenotypes in *H. volcanii*. DNA degradation for “food” is only one useful physiological function of a surface-bound nuclease in the DNA-rich milieu in which prokaryotes live. eDNA has been proposed as a structural element of bacterial biofilm structure (Dominiak et al., 2011; Godeke et al., 2011b), and plays additional roles within a biofilm such as aiding in attachment (Harmsen et al., 2010), self-organization (Gloag et al., 2013), and counteraction of antibiotic action (Chiang et al., 2013). It is not surprising then that extracellular nucleases in bacteria (including Hvo_1477 homolog Nuc, Table 2) modulate biofilm development (Kiedrowski et al., 2011). For example, the nucleases Dns in *Vibrio cholerae* and ExeM in *S. oneidensis* are involved in both eDNA processing for nutrition (ExeM) and/or natural transformation (Dns) and biofilm regulation (Blokesch and Schoolnik, 2008; Godeke et al., 2011a; Seper et al., 2011). Halobacteria form biofilms and like bacterial biofilms, high levels of eDNA are found in archaeal biofilms (Frols et al., 2012). It is possible then that Hvo_1477 is also involved in biofilm lifecycle through its putative activity as a surface-bound nuclease (Table 5).

## Author contributions

Scott Chimileski, Uri Gophna, Kunal Dolas, Adit Naor, and R. Thane Papke conceived of the research and designed the experiments. Scott Chimileski, Kunal Dolas, and Adit Naor carried out and analyzed the experiments, and Scott Chimileski, Uri Gophna, Kunal Dolas, Adit Naor, and R. Thane Papke wrote the manuscript.

### Conflict of interest statement

The authors declare that the research was conducted in the absence of any commercial or financial relationships that could be construed as a potential conflict of interest.
